# Effects of antidiabetic drugs on bone metabolism

**DOI:** 10.1515/almed-2024-0038

**Published:** 2024-04-03

**Authors:** Nuria Padilla Apuntate, Carmen G. Puerto Cabeza, Alba Gallego Royo, Nuria Goñi Ros, Claudia Abadía Molina, Javier Acha Pérez, Pilar Calmarza

**Affiliations:** Service of Clinical Biochemistry, Miguel Servet University Hospital, Zaragoza, Spain; Service of Preventive Medicine, Miguel Servet University Hospital, Zaragoza, Spain; Service of Endocrinology and Nutrition, Miguel Servet University Hospital, Zaragoza, Spain; Institute of Biomedical Research (IIS) of Aragón, Zaragoza, Spain; University of Zaragoza, Zaragoza, Spain; Spanish Network-Center for Cardiovascular Biomedical Research) (CIBERCV), Carlos III Health Institute, Madrid, Spain; Member of SEQCML Oxidative Stress Commission and Lipoproteins and Vascular Diseases Commission, Madrid, Spain

**Keywords:** diabetes mellitus type 2, bone remodeling markers, antidiabetic drugs, pioglitazone

## Abstract

**Objectives:**

The prevalence of diabetes mellitus type 2 (DMT2) is increasing exponentially worldwide. DMT2 patients have been found to be at a higher risk for bone fractures than the healthy population. Hence, improving our understanding of the impact of antidiabetic drugs on bone metabolism is crucial.

**Methods:**

A descriptive, retrospective study involving 106 patients receiving six groups of antidiabetic drugs: insulin; dipeptidylpeptidase four inhibitors (DPP4i); glucagon-like peptide type 1 receptor agonists (GLP1ra); sulfonylureas; sodium-glucose cotransporter two inhibitors (SGLT2i); and pioglitazone, in which osteocalcin (OC), bone alkaline phosphatase (BAP) and C-terminal telopeptide of collagen type 1 or beta-crosslaps (β-CTx) were determined.

**Results:**

β-CTx concentrations were higher in the patients treated with pioglitazone, as compared to patients treated with DPP4i (p=0.035), SGLT2i (p=0.020) or GLP1ra (p<0.001). The lowest β-CTx concentrations were observed in the patients treated with GLP1ra.

**Conclusions:**

Bone remodeling is influenced by the type of antidiabetic drug administered to DMT2 patients. In our study, the patients who received pioglitazone showed higher β-CTx concentrations, as compared to patients treated with other types of antidiabetic drugs. This finding highlights the convenience of avoiding these drugs, especially in postmenopausal women with DMT2. GLP1ra drugs were associated with the lowest β-CTx concentrations, which suggests that these agents could exert beneficial effects on bone metabolism.

## Introduction

Diabetes mellitus affects 422 million people worldwide. In the recent years, the prevalence of this disease has increased exponentially, especially in low and middle-income countries [1]. The most common form is diabetes mellitus type 2 (DMT2), which accounts for 90 % of cases and is characterized by insulin resistance, often associated with relative insulin deficiency [2].

Patients with DMT2 are at a higher risk for fragility fractures, as compared to the healthy population [3]. In contrast with fractures associated with primary osteoporosis, these patients show normal or even elevated bone mineral density (BMD) values but poor bone resistance. This low bone resistance may be due to abnormal bone microarchitecture and remodeling, added to defective mineralization. Other factors contributing to poor bone resistance include increased oxidative stress and inflammation [4].

The bone is a dynamic tissue that undergoes constant remodeling throughout life. This process entails the resorption of old bone followed by the formation of new bone, to preserve biomechanical competence and mineral homeostasis, thereby preventing bone lesions. The slowing of bone remodeling leads to the formation of bone tissue with normal/high mineral density but with a higher risk for fractures. Such is the case of DMT2 patients, who experience a reduction of bone remodeling [5], due to the direct and indirect effects of hyperglycemia, hyperinsulinemia during the early phases of the disease, obesity, and the effect of oral antidiabetic drugs.

Some histomorphometric studies reveal that DMT2 patients have a reduced number of osteoblasts [6] and a meta-analysis of 22 studies involving patients with DMT2 demonstrated that bone remodeling was lower in these patients, with low concentrations of osteocalcin (OC) (bone formation marker) and beta crosslaps (β-CTx), bone resorption marker, as compared to healthy controls [7]. These results were confirmed by a recent meta-analysis [8].

However, although we have appropriate methods for evaluating bone remodeling markers (BRM) in serum [9] and even though the potential association between reduced bone remodeling and an increased risk for fragility fractures in DMT2 patients, there is no enough evidence that bone remodeling markers are predictive of the occurrence of fractures in these patients [5]. Additionally, the measurement of bone mineral density (T-score measurement) and the use of the Fracture Risk Assessment Tool (FRAX) may underestimate the prediction of fractures in patients with diabetes, especially in DMT2 patients.

The impact of antidiabetic drugs on bone mineral density has consistently been demonstrated in a variety of studies of the recent decades [10, 11] and may contribute to an abnormal bone remodeling in DMT2 patients and to an increased risk for fractures in this already vulnerable population. Fragility fractures in diabetic patients may be very severe and can cause disability or even death.

The objective of this study was to determine the effect of antidiabetic drugs on bone remodeling. To such purpose, measurements of bone formation (OC and BAP) and bone resorption markers (β-CTx) were performed in a sample of the population in our health district.

## Materials and methods

A descriptive, retrospective study was performed in 106 patients (36.8 % women, all of them postmenopausal) with a mean age of 67±8 years, affected by DMT2 and receiving antidiabetic therapy. All patients underwent a blood test, which included BRM, between May 2020 and February 2021. The selection of the study population was based on similar ages, a balanced men-to-women ratio and was subdivided into six groups, according to the antidiabetic therapy received. All patients also received metformin.

The groups established, according to treatment were as follows:

– Group 1: Insulin: 15 patients, median age (MA): 68 years (Q1–Q3: 61–78 years).
– Group 2: DPP4i: 28 patients, MA: 68 years (Q1–Q3: 66–72 years).
– Group 3: GLP1ra: 16 patients, MA: 64 years (Q1–Q3: 58–73 years).
– Group 4: Sulfonylureas: 16 patients, MA: 66 years (Q1–Q3: 58–71 years).
– Group 5: SGLT2i: 15 patients, MA: 68 years (Q1–Q3: 59–70 years).
– Group 6: Thiazolidinediones (pioglitazone): 16 patients, MA: 68 years (Q1–Q3: 62–73 years).

Patients with comorbidities or receiving any other drug therapy that could interfere with bone metabolism were excluded. None of the patients were supplemented with vitamin D. The minimum duration of treatment was≥3 years.

This study complies with all national regulations, institutional policies and the ethical principles of the Declaration of Helsinki and was approved by the Ethics Committee of scientific research of the Autonomous Community of Aragon (CEICA).

Blood samples were drawn early in the morning, after eight-hour overnight fasting. Following serum separation, the samples were stored at −80 °C for later single-run analysis. No specific dietary instructions were provided, and all patients followed their usual diet.

OC, BAP and β-CTx were measured in all patients. OC and β-CTx were determined by enzyme immunoassay (N-MID Osteocalcin ELISA, Inmunodiagnostic System Ltd, Boldon, UK and Serum Crosslaps, Inmunodiagnostic Systems Ltd, Boldon, UK), respectively, as well as BAP (Microvue BAP, EIA, Quidel corporation, San Diego, CA, USA). The following ranges of reference were used: β-CTx (115–684 pg/mL); OC (5.8–39.8 ng/mL); and BAP (12–43 IU/L). The detection thresholds for OC, BAP and β-CTx were 0.5 ng/mL, 0.7 U/L, and 0.020 ng/mL, respectively. Intra-assay coefficients of variation did not exceed 2.2 ng/mL, 5.8 U/L and 3 ng/mL, respectively. Inter-assay coefficients of variation did not exceed 5.1 ng/mL, 7.6 U/L and 10.9 ng/mL, respectively, for each of the parameters indicated.

All statistical analyses were performed with IBM SPSS^®^ Statistics 21.0 (IBM Corporation, Armonk, NY). The global normality of all the parameters studied was examined by Kolmorogov Smirnoff test. Age, weight, and height were found to be normally distributed, whereas BMI, age at DMT2 onset, glycated hemoglobin (HbA_1c_), OC, BAP, and β-CTx, were not normally distributed. To check the normality of these parameters, according to the pharmacological group administered, the test of Shapiro-Wilk (group sample size<50) was used. It was observed that all these parameters do not met normal conditions in any of the pharmacological groups, therefore, non-parametric tests have been used for its study.

To check if there were differences in each of the parameters mentioned in the different groups of patients, according to treatment, the Kruskall Wallis H test was used, completing the analysis with the Bonferroni test for multiple comparisons between categories, in those variables in which significant differences were obtained. A p value<0.05 was considered statistically significant. Potential sex-based differences across groups were examined by the Chi-square test. Spearman’s Rho test was used to determine correlations between quantitative variables.

## Results

Antrophometric data, glycemic control (HbA_1c_) and time since DMT2 onset, in the different patient groups, are shown in Table 1. No statistically significant sex-based differences were observed between different types of antidiabetic treatment (p=0.956). There were no significant differences in relation to age (p=0.193) or HbA_1c_ (p=0.218) concentrations. In contrast, significant differences were observed in weight, BMI, and time since DMT2 onset (p=0.002, p=0.001 and p=0.023, respectively). More specifically, differences in weight were noted between the DPP4i and the GLP1ra group (p=0.003) and between the pioglitazone group and the GLP1ra group (p=0.007). In relation to BMI, there were significant differences between the DPP4i and the GLP1ra group (p=0.001), and between the pioglitazone and the GLP1ra group (p=0.025), being BMI higher in patients treated with GLP1ra.

**Table 1:** Anthropometric data of the patients, years since onset of type 2 diabetes mellitus and HbA1c according to antidiabetic treatment received.

Antidiabetic treatment	Female sex, %	Age, years^a^	Weight, kg^a^	Height, cm^a^	BMI, kg/m^2a^	Years from DMT2 onset^a^	HbA_1c_, %^a^
Insulin n=15	46.7	68 (61–78)	77.6 (72.2–83.0)	161 (150–167)	29.8 (26.6–33.6)	13.0 (11.0–16.0)	8.10 (7.40–8.85)
DPP4 n=28	35.7	68 (66–72)	73.2 (71.0–78.9)	167 (158–171)	25.9 (24.9–29.5)	9.5 (8.0–11.0)	7.15 (6.50–7.50)
arGLP1 n=16	33.3	64 (58–73)	96.5 (85.0–99.8)	164.5 (158–171)	34.4 (31.2–35.4)	10.0 (7.5–11.0)	7.40 (6.95–8.10)
Sulfonylureas n=16	37.5	66 (58–71)	74.9 (66.9–90.0)	165 (158–167)	28.7 (21.3–26.1)	10.5 (10–16.3)	7.15 (6.65–7.90)
SGLT2i n=15	31.3	68 (59–70)	83.6 (77.0–92.3)	166 (159–171)	30.0 (27.9–32.7)	11.5 (8.8–13.3)	7.30 (7.00–7.92)
Pioglitazone n=16	40	68.5 (62–73)	73.5 (63.9–79.8)	160 (153–164)	27.1 (21.6–27.1)	11.0 (10.0–14.0)	7.30 (6.65–8.00)

^a^Results expressed as median and interquartile range. BMI, body mass index; T2DM, diabetes mellitus type 2; HbA_1c_, glycated hemoglobin; DPP4i, dipeptidylpeptidase inhibitors; GLP1ra, glucagon-like peptide type 1 receptor agonists; SGLT2i, sodium-glucose cotransporter type 2 inhibitors.

The patients receiving DPP4i were significantly older than those receiving insulin (p=0.025). All patients had normal kidney and liver function and had been receiving their antidiabetic therapy for at least 3 years.

**Figure 1:**
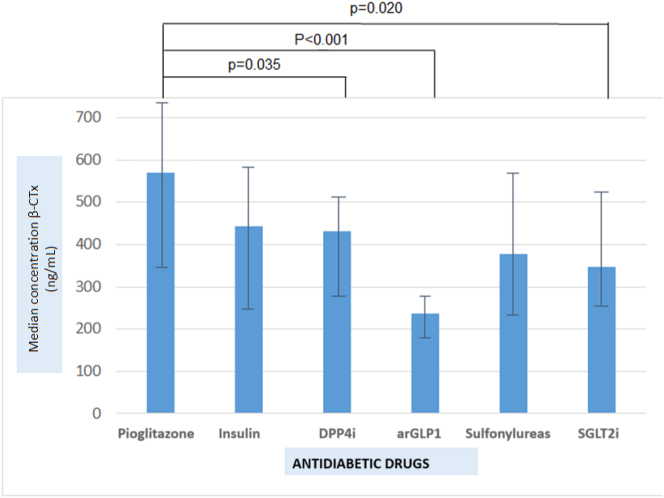
Differences in concentrations of bone remodelling markers (β-CTx) according to the antidiabetic treatment received. Bars indicate median and interquartile range. DPP4i, dipeptidylpeptidase 4 inhibitors; arGLP1, glucagon-like peptide type 1 receptor agonists; iSGLT2, sodium-glucose cotransporter type 2 inhibitors.

Statistically significant differences were observed in the following bone markers according to the pharmacological group: OC (p=0.026), BAP (p=0.007), and β-CTx (p=0.002) (Table 2). After Bonferroni test, differences persisted only for β-CTx, with values being higher in the pioglitazone group, as compared to the SGLT2i (p=0.020), DPP4i (p=0.035) and GLP1ra (p<0.001) group (Figure 1).

**Table 2:** Concentration of bone remodelling markers, according to the antidiabetic treatment received.

Antidiabetic treatment	Osteocalcin^a^, ng/mL	Bone alkaline phosphatase^a^, ng/mL	β-Crosslaps^a^, pg/mL
Insulin	8.2 (5.2–16.6)	31 (24.6–36.4)	456.6 (244.5–589.6)
DPP4i	5.9 (5.4–7.7)	28.5 (23.6–36.7)	436.2 (269.1–508.9)
arGLP1	6 (3.2–8.7)	22 (17.8–27.1)	239.8 (196.4–279.3)
Sulfonylureas	10 (5.8–12.5)	21.6 (18.4–26.8)	379.8 (232.8–565.8)
SGLT2i	5.4 (3.9–10.6)	22.4 (18.6–24.8)	349.1 (249.6–515.8)
Pioglitazone	10.6 (6.9–15.4)	23.3 (19.5–27.9)	572.9 (344.2–718)

^a^Results expressed as median and interquartile ranges. DPP4i, dipeptidylpeptidase inhibitors; GLP1ra, glucagon-like peptide type 1 receptor agonists; SGLT2i, sodium-glucose cotransporter type 2 inhibitors.

The insulin group, along with the sulfonylureas and pioglitazone group, exhibited the highest concentrations of bone formation markers.

In the case of DPP4i, BAP concentrations were higher, as compared to other groups. In contrast, OC concentrations were lower, as compared to other groups.

In relation to markers of bone resorption, the patients in the GLP1ra group were the ones that showed the lowest β-CTx concentrations, followed by the SGLT2i and sulfonylureas group. The highest β-CTx concentrations were observed in the patients receiving pioglitazone (Table 2).

A significant, positive correlation was observed between OC and BAP (Spearman’s Rho 0.346, p=0.001); between OC and β-CTx (Spearman’s Rho 0.552 p<0.001), and between BAP and β-CTx (Spearman’s Rho 0.298 p=0.003). In relation to the association between bone markers according to the type of antidiabetic treatment received there was a significant positive correlation between OC and β-CTx (Spearman’s Rho 0.604, p=0.017) in the insulin group. A significant positive correlation was also observed between β-CTx and BAP (Spearman’s Rho 0.476, p=0.064) in the group of patients treated with DPP4i.

In the SGLT2i group, β-CTx correlated positively with OC (Spearman’s Rho 0.656, p=0.006). In the pioglitazone group, BAP correlated positively with OC (Spearman’s Rho 0.531, p=0.042), and β-CTx correlated positively with OC (Spearman’s Rho 0.531, p=0.042). In the groups of patients treated with GLP1ra and sulfonylureas no significant correlation was observed between the different bone markers.

## Discussion

The results of our study indicate an association between GLP1ra and a low bone turnover, with values for markers of bone formation and resorption being lower in this group. In contrast, insulin and pioglitazone were found to be associated with a substantially higher bone turnover, expressed as higher levels of the two markers of bone remodeling.

The results obtained for the insulin group are consistent with some previous studies [12, 13] revealing that insulin exerts a significant anabolic effect on osteoblasts. However, these results are not supported by Ivaska et al. [14], who found that insulin reduces β-CTx and OC concentrations, also in contrast to our results.

Kanazawa et al. [15] documented that insulin was associated with a higher risk for fractures, but Napoli et al. [16] and Hidayat et al. [17] suggested that this association could be explained by an increase in the frequency of severe hypoglycemic events, which results in a higher risk for falls.

DPP4i and GLP1ra are considered to have beneficial effects on bone metabolism, as they increase osteoblast function and reduce osteoclast activity, but these results are not supported by other authors such as Yang et al. [18], who suggested that an increase in DPP4i activity may indirectly promote bone resorption and inhibit bone formation. However, a recent study [19] revealed reduced levels of OC and increased TRAP5b concentrations in mice models of DMT2 and these values were reversed after the administration of linagliptin (DPP4i). Abdi et al. [20] used two Wistar mice models of DMT2 treated with DPP4i and GLP1ra, respectively, observed that the two therapies reduced bone resorption, with the mice treated with GLP1ra showing a more favorable bone profile. These findings are in agreement with our study, as concentrations of bone resorption markers were lower in the group of patients treated with GLP1ra.

With respect to SGLT2i, Dong et al. [21] found that canagliflozin induced an increase in bone resorption (β-CTx) and bone formation (OC) concentrations. In contrast, although dapagliflozin and empagliflozin (of the SGLT2 group too) did not have any significant effects on bone formation markers, dapagliflozin led to a significant increase in bone resorption, which indicates deleterious effects on bone mineral density. Jackuliak et al. [22] suggested that the group of SGLT2 antidiabetics has neutral effects on bone markers. This finding is supported by the results obtained by Tang et al. [23], who did not observe a higher risk for fractures in patients receiving SGLT2i. In our study, the patients treated with SGLT2i exhibited intermediate OC and β-CTx values.

With regard to sulfonylureas, Jackuliak et al. [22] pointed that this group of patients has a similar risk for fracture than other groups, although with increased bone formation and reduced bone resorption. Our study provided similar results, as OC concentrations exceeded the median for all groups, whereas β-CTx fell below the median value.

In our study, the highest concentrations of bone resorption markers were observed in the pioglitazone group, as compared to the other pharmacological groups. In fact, only the differences found initially were maintained in the case of β-CTx, with higher concentrations.

In the patients treated with pioglitazone, as compared to patients treated with SGLT2i, DPP4i and GLP1ra. Differences were more substantial between the pioglitazone and the GLP1ra group.

Some authors consistently report that this agent induces an increase in bone resorption, whereas others not. So, Mori et al. [24] reported that pioglitazone increases bone resorption, regardless of age and sex, whereas Xiao et al. [25] affirmed that pioglitazone inhibits bone formation without it affecting bone resorption.

The results of our study suggest that these drugs influence the bone resorption process. Thus, the patients treated with pioglitazone showed significantly higher concentrations of bone resorption markers, as compared to the other groups. Therefore, avoiding the use of these drugs should be considered, especially in postmenopausal women with DMT2.

However, GLP1ra drugs were associated with lower values of bone resorption markers, which could indicate a beneficial effect on bone metabolism.

In conclusion, the type of antidiabetic therapy received may influence bone remodeling and, as a result, bone health in patients with DMT2.
